# Experience-Related Changes in Place Cell Responses to New Sensory Configuration That Does Not Occur in the Natural Environment in the Rat Hippocampus

**DOI:** 10.3389/fphar.2017.00581

**Published:** 2017-08-23

**Authors:** Dan Zou, Hiroshi Nishimaru, Jumpei Matsumoto, Yusaku Takamura, Taketoshi Ono, Hisao Nishijo

**Affiliations:** ^1^System Emotional Science, Graduate School of Medicine and Pharmaceutical Sciences, University of Toyama Toyama, Japan; ^2^Department of Pathophysiology, Shenyang Medical College Shenyang, China

**Keywords:** hippocampus, place cell, sensory conflict, backward translocation, mismatch cell

## Abstract

The hippocampal formation (HF) is implicated in a comparator that detects sensory conflict (mismatch) among convergent inputs. This suggests that new place cells encoding the new configuration with sensory mismatch develop after the HF learns to accept the new configuration as a match. To investigate this issue, HF CA1 place cell activity in rats was analyzed after the adaptation of the rats to the same sensory mismatch condition. The rats were placed on a treadmill on a stage that was translocated in a figure 8-shaped pathway. We recorded HF neuronal activities under three conditions; (1) an initial control session, in which both the stage and the treadmill moved forward, (2) a backward (mismatch) session, in which the stage was translocated backward while the rats locomoted forward on the treadmill, and (3) the second control session. Of the 161 HF neurons, 56 place-differential activities were recorded from the HF CA1 subfield. These place-differential activities were categorized into four types; forward-related, backward-related, both-translocation-related, and session-dependent. Forward-related activities showed predominant spatial firings in the forward sessions, while backward-related activities showed predominant spatial firings in the backward sessions. Both-translocation-related activities showed consistent spatial firings in both the forward and backward conditions. On the other hand, session-dependent activities showed different spatial firings across the sessions. Detailed analyses of the place fields indicated that mean place field sizes were larger in the forward-related, backward-related, and both-translocation-related activities than in the session-dependent activities. Furthermore, firing rate distributions in the place fields were negatively skewed and asymmetric, which is similar to place field changes that occur after repeated experience. These results demonstrate that the HF encodes a naturally impossible new configuration of sensory inputs after adaptation, suggesting that the HF is capable of updating its stored memory to accept a new configuration as a match by repeated experience.

## Introduction

The hippocampal formation (HF) is involved in encoding and retrieval of episodic memory (Squire et al., 1993; Schacter et al., 1996; Vargha-Khadem et al., 1997; Frank et al., 2000; Wood et al., 2000; Yancey and Phelps, 2001; Ferbinteanu and Shapiro, 2003; Eichenbaum, 2004). Previous lesion and neurophysiological studies using rodents and humans suggest that the HF does not encode new sensory stimuli themselves, but does encode new temporal or spatial combinations of each sensory stimulus (Honey et al., 1998; Wan et al., 1999; Eichenbaum, 2000; Takakura et al., 2003; Furusawa et al., 2006). Consistent with these findings, recent studies suggest that, to encode new information, the HF may function as a comparator to detect differences (i.e., mismatch) between internal representation in the HF and actual sensory inputs from the environment (Gray, 1982; Hasselmo and Schnell, 1994; Hasselmo and Wyble, 1997; Moser and Paulsen, 2001; Vinogradova, 2001; Havekes and Timmer, 2007; Kumaran and Maguire, 2007; Takahashi and Sakurai, 2009; Zou et al., 2009). Thus, the HF holds internal representation of the environmental information that will be compared with incoming information (Gray, 1982; Vinogradova, 2001; Kumaran and Maguire, 2007). Consistently, the environmental information is stored as a map-like representation in the HF (O’Keefe and Nadel, 1978). Place cells, the activity of which increases in a specific location in the environment, may represent this information (O’Keefe and Dostrovsky, 1971; McNaughton et al., 1983; Eichenbaum et al., 1987; Wiener et al., 1989; Kobayashi et al., 1997). Furthermore, previous neurophysiological studies reported that the HF place-related neurons represented configuration of various information encountered during navigation (Ono et al., 1993; Dayawansa et al., 2006; Ho et al., 2008; Hori et al., 2011). Another type of HF neurons has been reported (mismatch cells). Mismatch cells are active when the rats find a novel stimulus or fail to find a familiar stimulus at a particular location (O’Keefe and Nadel, 1978; Otto and Eichenbaum, 1992). These two types of the HF neurons might be important components in the HF neural circuits for a comparator that detects sensory mismatch.

Sensory mismatch has been implicated in motion sickness (Kohl, 1983; de Graaf et al., 1998). Vestibular organs including semicircular canals and otolith organs play an important role to induce motion sickness (Igarashi, 1990). When convergent sensory-motor inputs including vestibular, visual, and somatosensory inputs as well as motor efferent copies do not match the expected sensory patterns in the HF store, spatial orientation is disturbed, inducing motion sickness. Unit recording studies in zero-gravity parabolic flight and in the Space Shuttle reported that activity of HF place cells as well as head direction cells in the thalamus, which sends directional information to the HF, was abnormal in such environment, where humans often suffer from motion sickness (Knierim et al., 2000, 2003; Taube et al., 2004). Furthermore, previous behavioral studies with pharmacological manipulation in the HF suggest that the HF is involved in motion sickness in rodents (Horii et al., 1994; Uno et al., 2000). These findings suggest that this neural mismatch signal may be generated in the HF. Furthermore, training in mismatch condition ameliorates motion sickness (Stern et al., 1989; Stroud et al., 2005). This further suggests that the neural store in the HF is also updated by the neural mismatch signals to register a new configuration of sensory-motor inputs. When the neural store in the HF is updated, the HF comparator accepts the mismatch condition as the match, and the learning (i.e., adaptation/habituation) processes are terminated (Takeda et al., 2001).

Previous studies suggest that theta rhythm is implicated in both the encoding and retrieval of information in the HF (Hasselmo et al., 2002; Manns et al., 2007b; Terada et al., 2017) and also in modulation of HF synaptic current during learning (Brankack et al., 1993; Wyble et al., 2000; Orr et al., 2001), suggesting that HF theta waves might reflect the activity of a HF comparator during navigation (Zou et al., 2009). In our previous studies, to analyze HF theta rhythm and thalamic head direction cell activity in a mismatch condition, rats locomoted on a treadmill that was translocated along a figure 8-shaped track by a motion stage (Zou et al., 2009; Enkhjargal et al., 2014). In a mismatch condition, although rats locomoted forward on the treadmill, the treadmill itself was translocated backward (Zou et al., 2009; Enkhjargal et al., 2014). In this mismatch condition, idiothetic sensory inputs (optic flow, vestibular inputs, and proprioceptive inputs or motor efferent copies) contradicted each other (i.e., mismatched); movement direction indicated by the proprioceptive inputs and/or motor efferent copies during locomotion did not match that indicated by the visual-vestibular inputs. Both types of information (i.e., locomotion-related and vestibular inputs) are reported to be indispensable for HF activity (Hirase et al., 1999; Dayawansa et al., 2006; Russell et al., 2006; Lu and Bilkey, 2009). The results in the mismatch condition indicated that sensory conflict (mismatch) among idiothetic sensory inputs elevated HF theta activity, while theta activity gradually decreased after repeated exposure to the conflict (Zou et al., 2009). This suggests that new place cells encoding a new configuration of convergent inputs to the HF are formed in the HF after repeated experience.

Among the HF neural circuits, those in the CA1 area play a critical role in stimulus encoding as well as retrieval (Manns et al., 2007b), suggesting that a new configuration of convergent inputs could be represented in this area. Consistent with the idea, a recent neurophysiological study reported that some HF CA1 neurons showed specific spatial responses only during backward ambulation, but not during forward ambulation, while some other HF CA1 neurons showed spatial responses only during forward ambulation (Maurer et al., 2014). However, another recent study reported that HF CA1 neurons showed similar spatial responses during forward and backward translocation on a treadmill (Cei et al., 2014). The difference between these two studies might be ascribed to the difference in movement patterns of extremities between the two studies (reverse ambulation vs. forward locomotion on a treadmill during backward translocation) (Cei et al., 2014). However, there might be another possibility; the rats were well-trained with backward ambulation in the former study (Maurer et al., 2014), while the rats were trained only in the forward condition in the latter study (Cei et al., 2014). Therefore, we hypothesized that the differences in spatial responses between the two studies might be attributed to the differences in experiences (training) in the backward condition, and that HF neurons would develop different spatial responses between the forward and backward translocation on a treadmill after being well-trained.

In the present study, to investigate whether the HF CA1 place cells were capable of encoding mismatched information that does not occur naturally after being well-trained, we recorded the HF CA1 neuronal activity from rats that were well-trained in a set-up that replicated the mismatch conditions of the previous study (Zou et al., 2009).

## Materials and Methods

### Subjects

Seven Wistar male rats weighing 200–300 g were used. The animals were similarly treated according to our previous protocols (Dayawansa et al., 2006; Zou et al., 2009; Enkhjargal et al., 2014). They were individually housed in cages controlled at a constant temperature (20 ± 2°C) with free access to water and laboratory chow. All rats were treated in strict compliance with the United States Public Health Service Policy on the Humane Care and Use of Laboratory Animals, the National Institutes of Health Guide for the Care and Use of Laboratory Animals, and the Guidelines for the Care and Use of Laboratory Animals of the University of Toyama. The study was approved by the Committee for Animal Experiments and Ethics at the University of Toyama (Permit number: S-2009MED-25).

### Surgery

The same surgical procedures were used as those in our previous studies (Dayawansa et al., 2006; Zou et al., 2009; Enkhjargal et al., 2014). Briefly, the rats were anesthetized with pentobarbital sodium (40 mg/kg, i.p.). First, several stainless screws were implanted in the bone as anchors. One of the screws over the cortex near the HF was used as a ground electrode. Then, a cranioplastic cap was molded on the skull according to our previous studies (Nishijo and Norgren, 1990; Uwano et al., 1995). After the surgery, an antibiotic (gentamicine sulfate) was administered topically and systemically (2 mg, i.m.). This cranioplastic cap was used as artificial earbars; the cranioplastic cap can be painlessly fixed in the stereotaxic apparatus on the treadmill. After 1 week of recovery, the rats were trained in a navigation task on a treadmill (see “Training and behavioral testing” in detail).

After training, the rats were reanesthetized, and a hole (3–5 mm diameter) for semi-chronic recording was drilled through the cranioplastic and the underlying skull over the cortex near the HF (A, -2.0 to -4.0 from bregma: L, 2.0 to 6.0). The exposed dura was removed, and the hole was covered with a sterile Teflon sheet and sealed with epoxy glue for later neuronal recording.

### Experimental Setup and Tasks

**FIGURE 1:**
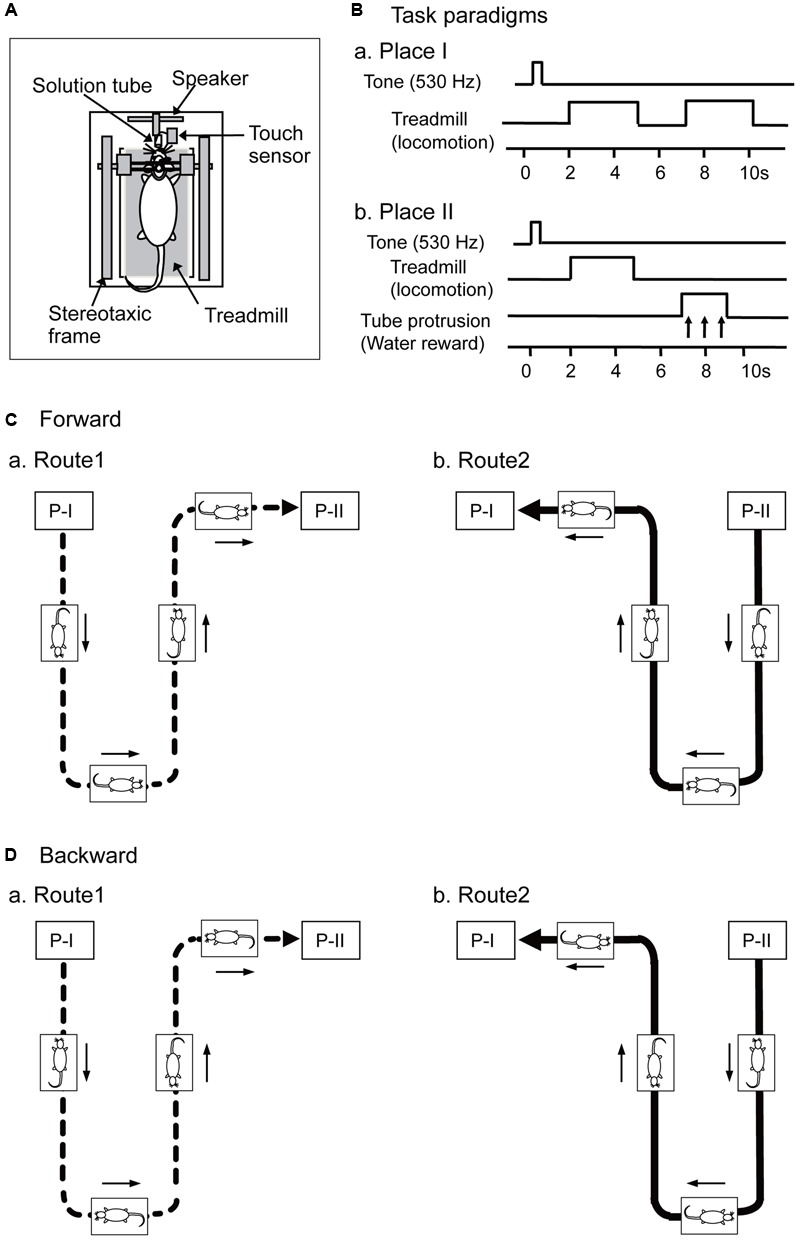
Schema of the experimental paradigms. **(A)** Set up of a stereotaxic apparatus and a treadmill that were attached to a motion stage, and the rat was placed on the treadmill inside a spacious transparent plastic enclosure. **(B)** Paradigms of a delayed stimulus-response association (DSR) task that was carried out at Places I and II. At Place I **(Ba)**, the task was initiated by a cue tone and followed by two periods of 3.0 s during which the treadmill rotated. At Place II **(Bb)**, the task was initiated by the same cue tone and followed by a 3.0 s period of treadmill rotation and a 2.0 s period of tube protrusion. The tone and the following treadmill operation were separated by 1.5 s intervals, while the two reinforcements were separated by 2.0 s intervals. **(C,D)** Movements of the motion stage in forward (**C**, forward sessions) and backward translocation (**D**, backward sessions). The motion stage moved on a Figure 8-shaped route consisting of Routes 1 (a) and 2 (b), and the start point of each route was designated as Places I and II, respectively. Arrows indicate movement direction of the motion stage.

The same apparatus and tasks were used as those in our previous studies (Dayawansa et al., 2006; Zou et al., 2009; Enkhjargal et al., 2014). Briefly, a transparent plastic enclosure for semi-chronic recording (Nishijo and Norgren, 1990) was placed on a treadmill, which was fixed on a stereotaxic apparatus. The enclosure lacked a floor so that the rats could locomote on the treadmill. The stereotaxic apparatus was further attached to the motion stage (**Figure 1A**). The cranioplastic cap on the rat’s head was painlessly fixed to a stereotaxic frame on the motion stage. The motion stage was translocated horizontally by belts with two motors (THK Co., Kanazawa, Japan). Another motor, attached to the base of the motion stage, rotated the motion stage so that the rat faced in the direction of translocation.

In a forward condition (**Figure 1C**), the motion stage was translocated between Places I and II in a figure 8-shaped pathway consisting of Routes 1 and 2 at the speed of 20 cm/s. During this translocation, the rats always faced toward the direction of the tangent of the translocation routes to imitate directional changes in natural navigation. The treadmill was also operated at the same speed (20 cm/s) as the translocation speed of the stage, which reliably induced locomotion of the rat. In Route 1 (**Figure 1Ca**), the rat was translocated from Place I to Place II, and in Route 2 (**Figure 1Cb**), from Place II to Place I. Thus, Routes 1 and 2 included a common central stem in the figure 8-shaped pathway. At the Places I and II, the motion stage paused, and a delayed stimulus-response association (DSR) task was imposed (see below in detail). After the DSR task, the motion stage was rotated so that the rat faced in the direction of translocation in Routes 1 and 2 before translocation.

In a different condition (backward condition), the motion stage was initially rotated by 180°, and then translocated in the same way (**Figure 1D**). In this backward condition, although the rat locomoted forward on the treadmill, the motion stage was translocated backward (opposite to the rat’s locomoting direction) (i.e., mismatch condition).

In both the forward and backward conditions, the stage stopped at the end of the pathways (i.e., Places I and II), where the rats were required to perform the DSR task (**Figures 1Ba,b**). In the DSR task, the rats could acquire rewards, which was important to keep the rats locomoting on the treadmill throughout the recording sessions. At Place I, the DSR task started by the 530-Hz tone for 0.5 s (**Figure 1Ba**). After a 1.5 s delay, the treadmill was operated at 20 cm/s for 3.0 s twice with an intervening 2.0 s interval in which the treadmill was stopped. The rats reliably locomoted without reward during these 3.0 s runs. At Place II, the task similarly started by the same 530-Hz tone, and after a 1.5 s delay the treadmill was operated at 20 cm/s for 3.0 s (**Figure 1Bb**). After the second delay of 2.0 s, the tube was protruded close to the rat’s mouth for 2.0 s. The rat could ingest a water reward, if it licked the tube during this period. Water licking was detected by a touch sensor connected to the tube.

### Training and Behavioral Testing

The same training procedures were used as those in our previous studies (Zou et al., 2009; Enkhjargal et al., 2014). Briefly, the rats were acclimated to being placed for short periods in the plastic restraining enclosure on the motion stage before and after the surgery. Then, the rats were trained to perform the DSR task under a 24-h water-deprivation regimen. Second, after the rats could constantly perform the DRS task, they were trained to locomote on the treadmill while the motion stage was translocated in the forward condition for 2 weeks. The rats usually ingested 20–30 ml of water in the restrainer. If the rat failed to drink a total volume of 30 ml water, it was allowed to drink the remainder in its home cage. Our previous studies indicated that the rats well learned the tasks under the similar water-deprivation regimens (Dayawansa et al., 2006; Zou et al., 2009; Enkhjargal et al., 2014).

Finally, the rats were trained in the forward and backward conditions for 5 days. In this final training, a total of three sessions/day were conducted. Each session consisted of three laps of translocation. After each lap (Routes 1 and 2) of translocation, each three trials of the DSR task were imposed at Places I and II, respectively. In the 1st and 3rd sessions, the rats performed the task in the forward condition, while they did the task in the backward condition in the 2nd session. After these trainings, activity of the neurons was recorded from the HF CA1 area under the same protocol with three sessions; (1) an initial control session in the forward condition, in which both the stage and the treadmill moved forward, (2) a backward (mismatch) session, in which the stage was translocated backward while the rats locomoted forward on the treadmill, and (3) the second forward condition.

### Neuronal Recordings and Unit Isolation

The same neurophysiological procedures were used as those in our previous study (Dayawansa et al., 2006). Briefly, after the rat was placed in the stereotaxic apparatus on the motion stage, the Teflon sheet was removed, and a glass-insulated tungsten microelectrode (*Z* = 1.0–1.5 MΩ at 1 KHz; tip diameter < 5 μm) was stereotaxically inserted into various parts of the HF CA1 area. The neuronal signals, triggers for the tone, tube protrusion, and licking, and the *X*–*Y* coordinates of the motion stage were digitized and stored in a computer. The Offline Sorter program (Plexon, Dallas, TX, United States) sorted neuronal activities into single units by their waveform components. Superimposed wave forms of the sorted units were inspected to check the invariability of the sorted units throughout the recording sessions (see below in detail). Then, the sorted unit activities were transferred to the NeuroExplorer program (Nex Technology, Littleton, MA, United States) for further analysis.

Examples of superimposed spike waves of a HF neuron and the autocorrelograms of the neuronal spikes are shown in Supplementary Figure 1. We carefully inspected data throughout the sessions and those before and after the sessions. The data indicated that the superimposed waveforms (a) and various waveform parameters in cluster cutting projections (data not shown) were similar across the sessions. The autocorrelograms (b) showed that a refractory period of the CA1 neuron was 2–3 ms throughout the recording sessions, suggesting that these spikes were recorded from a single neuron.

When HF neuronal activities were isolated, their activities were recorded while the rats performed the task in the three sessions (see “behavioral testing” in detail). In these experimental conditions, effects of sensory mismatch were analyzed. Every condition always started at Place I in Route 1.

### Analysis of Place-Differential Activity

Place-differential activity was analyzed according to our previous study that used the same experimental setup and tasks (Dayawansa et al., 2006). Briefly, each route was divided into 56 successive pixels, and the firing rate maps in Routes 1 and 2 were separately constructed in each session. Place-differential activities were separately defined in Routes 1 and 2. First, the firing rate maps in each route were created by a smoothing method, in which the smoothed firing rate of a given pixel was defined as the mean of three pixels (the given pixel and the two adjoining pixels) (Dayawansa et al., 2006). Second, all pixels with an increase in the mean firing rate, which was defined as a firing rate greater than 2.0 times the grand mean firing rate of a given neuron in either Routes 1 or 2, were identified (Muller and Kubie, 1987; Kobayashi et al., 1997; Dayawansa et al., 2006). A place-differential activity was classified as such if it had at least three adjacent pixels with an increase in the mean firing rate (i.e., place field). This place cell analysis was separately carried out in individual sessions; 1st forward, 2nd backward, and 3rd control sessions. Only the place-differential activities with place field(s) in at least one of the three sessions were further analyzed.

To assess changes in spatial firing patterns across the sessions, we computed pixel-to-pixel correlation coefficients (*r*) of firing-rate distributions between the first (control) and the following sessions (Dayawansa et al., 2006).

### Classification of Place-Differential Activities

The place-differential activities were initially classified based on the place fields and peak firing rates within the place fields. When the HF neuronal activities displayed place fields in the backward sessions and if the peak firing rates in the place fields of the backward session were more than four times of those in the control sessions in corresponding route, the activities were defined as backward-related regardless of correlation coefficients. When the HF neuronal activities displayed place fields at least in the 1st and/or last control sessions and if the peak firing rates in the place field(s) of the forward sessions were four times larger than those in the backward sessions in corresponding route, the activities were defined as forward-related regardless of the correlation coefficients in this route. The remaining place-differential activities that displayed place fields in both the forward and backward sessions were defined as both-translocation-related activities.

These forward-related and both-translocation-related activities were further grouped into two subcategories based on correlation coefficients. For forward-related activities, if correlation coefficients between 1st and 3rd forward sessions in a given route (Route 1 or Route 2) were larger than 0.4, the place-differential activities were defined as session-independent for that route. For both-translocation-related activities, if correlation coefficients between 1st and 3rd forward sessions and those between the 1st forward and 2nd backward sessions in a given route (Route 1 or Route 2) were larger than 0.4, the place-differential activities were defined as session-independent for that route. If one of the correlation coefficients did not reach 0.4, those activities were defined as session-dependent activities.

### Analyses of the Place Fields

Previous studies reported experience-dependent asymmetric expansion of place fields (Mehta et al., 1997, 2000; Lee et al., 2004). We similarly analyzed the place field expansion. First, place field sizes were simply analyzed by counting number of pixels within the place fields. Second, to analyze the firing rate distribution of the place field, skewness of firing rate distribution within the place field was computed (Mehta et al., 2000). Third, since skewness does not necessarily refer to asymmetry of spatial distribution of the firing rates in terms of movement direction of the rats, asymmetry of firing rate distribution within the place field was analyzed in terms of movement direction of the rats. In this analysis, the location of the center of the mass of the firing rate distribution within the place field was computed. Then, the place field was divided into two parts by the center of the mass. The place field asymmetry index was defined as difference in the number of the pixels between the two parts (i.e., the number of the pixels in the first part of the place field along the movement direction minus the number of the pixels in the second part of the place field).

Place field sizes and place field asymmetry index were compared by one way analysis of variance (ANOVA) and following *post hoc* comparisons (Fisher LSD test; *p* < 0.05) among the four types of the place-differential activities (forward-related, backward-related, both-translocation-related, and session-dependent activities).

### Histology

The same histological procedures were used as those in our previous study (Dayawansa et al., 2006). Briefly, upon completion of all the experiments, each rat was anesthetized with pentobarbital and several small electrolytic lesions were stereotaxically made around the recorded sites. The rats were then perfused, and the brains were removed and cut into serial 50 μm frontal sections. The brain sections were stained with Cresyl Violet. All marking and stimulation sites were then carefully verified microscopically. Positions of place-differential activities were sterotaxically located on the real tissue sections in each animal. Finally the recording sites were re-plotted on the corresponding sections on the atlas of Paxinos and Watson (1986).

## Results

The 161 HF CA1 neurons (complex spike cells) were recorded under three conditions; (1) initial control sessions, in which forward translocation with locomotion and the tasks were imposed, (2) backward sessions, in which backward translocation with locomotion and the tasks were imposed and (3) the last control sessions. Each neuron was recorded for three complete laps of translocation during each session. Of the 161 neurons, 56 place-differential activities were recorded from the HF CA1 subfield.

The place differential activities were categorized into four types; forward-related, backward-related, both-translocation-related, and session-dependent (**Table 1**). Of these, all forward-related and both-translocation-related activities displayed place fields with high similarity (i.e., *r* > 0.4) between the first and last control sessions in Route1, Route 2, or both. All of the 4 types of the HF place-differential activities were recorded from each rat, and percentages of each activity type were not different among the rats (data not shown). Furthermore, HF neurons with these four types of activities did not show activity change during the DSR task (data not shown).

**Table 1:** Response characteristics of the 56 hippocampal formation (HF) place-differential activities in Routes 1 and/or 2.

Category	Route 1	Route 2
Forward-related	12	17
Backward-related	14	5
Both-translocati on-related	11	3
Session-dependent	18	27
No place fields	1	4

Total	56	56

*The table indicates the number of the HF activities, response characteristic of which matched the definition of each category in each route. Note that place-differential activities were separately defined in each route, and some HF neurons showed spatial activities in both of the routes in different or same categories.*

### Forward-Related Activities

In Route 1, 12 activities (21.4%) showed place fields with high correlation coefficients between the 1st and 3rd forward sessions, but did not show place fields in the 2nd backward session. However, 2 of them showed high correlation coefficients between the 1st control and 2nd backward sessions. In Route 2, 17 activities (30.4%) showed place fields with high correlation coefficients between the 1st and 3rd forward sessions, but did not show place fields in the 2nd backward session. The mean correlation coefficients of these 29 activities between the first and last control session [0.64 ± 0.03 (mean ± SEM)] was significantly larger than that between the 1st and 2nd sessions (-0.001 ± 0.05) (Wilcoxon signed rank sum test, *P* < 0.001).

**FIGURE 2:**
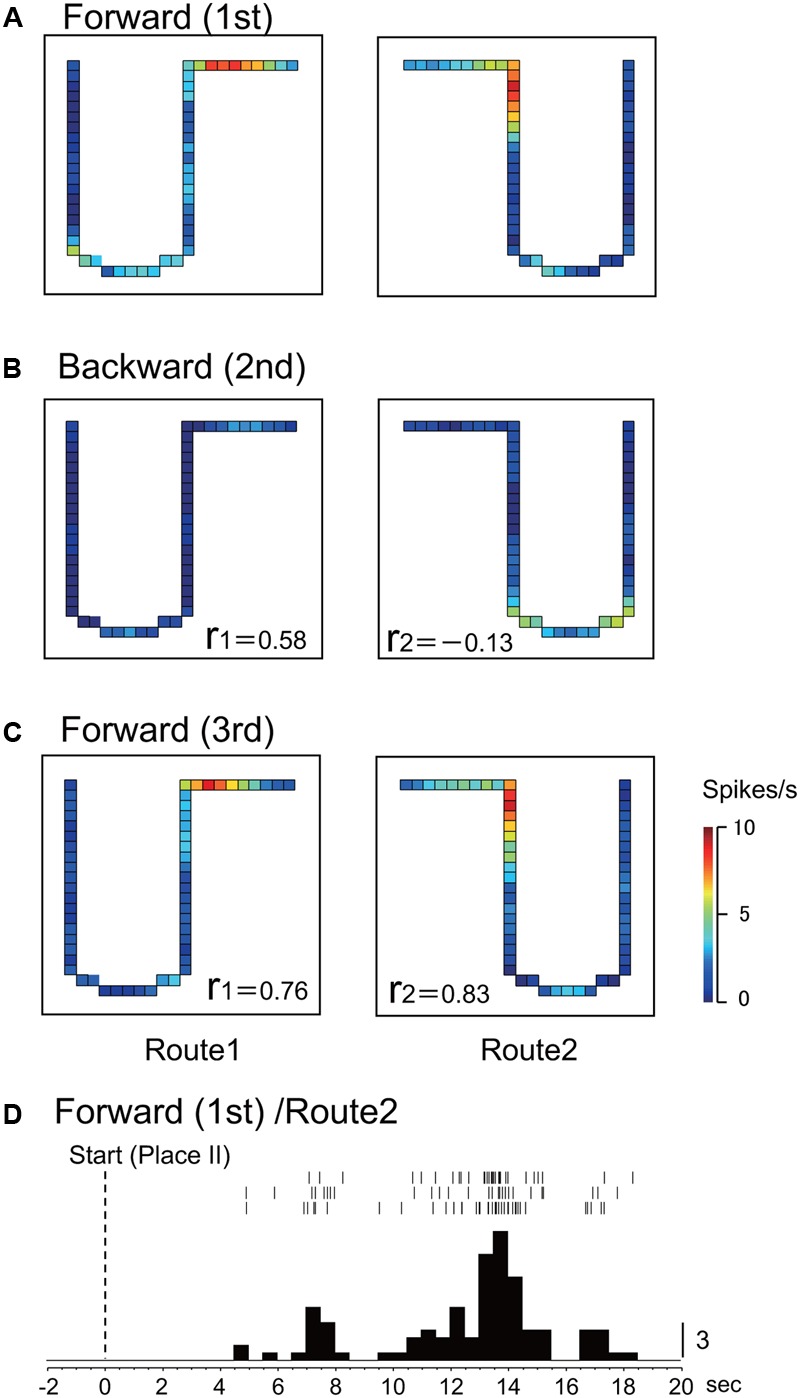
An example of a forward-related place-differential activity. **(A–C)** This activity displayed place fields in both Routes 1 and 2 in the 1st **(A)** and the 3rd **(C)** forward sessions, but not in the 2nd backward sessions **(B)**. *r*, correlation coefficients between firing-rate distributions in the first control session and the following given session in Route 1 (r 1) and Route 2 (r 2), respectively. The color of each pixel indicates neuronal activity calibrated at the right bottom (spikes/s). **(D)** Peri-event time histogram of the neuronal activity during forward translocation in Route 2 in the 1st session. The neuronal activity increased around 13 s after the start from Place II. A histogram shows summed neuronal activity. Each histogram bin, 500 ms; calibration at right of each histogram, number of spikes in each bin. The histograms were aligned with start of translocation.

**Figures 2A–C** shows the representative data of a HF place-differential activity that showed a spatial firing pattern dependent on forward translocation. This activity showed stable place fields in both the 1st and the last control sessions (forward translocation) on Route 1 (*r* = 0.76; 1st vs. 3rd) and Route 2 (*r* = 0.83; 1st vs. 3rd). It is noted that the peak firing rates in the place fields in Routes 1 and 2 decreased in the 2nd backward session to a level less than one fourth of those in the 1st control trials, although the correlation coefficient between the 1st and the 2nd sessions in Route 1 was relatively high (*r* = 0.58). An example of a peri-event time histogram of the neuronal activity during forward translocation in Route 2 in the 1st session is shown in **Figure 2D**. The neuronal activity increased around 13 s after the start from Place II.

### Backward-Related Activities

**FIGURE 3:**
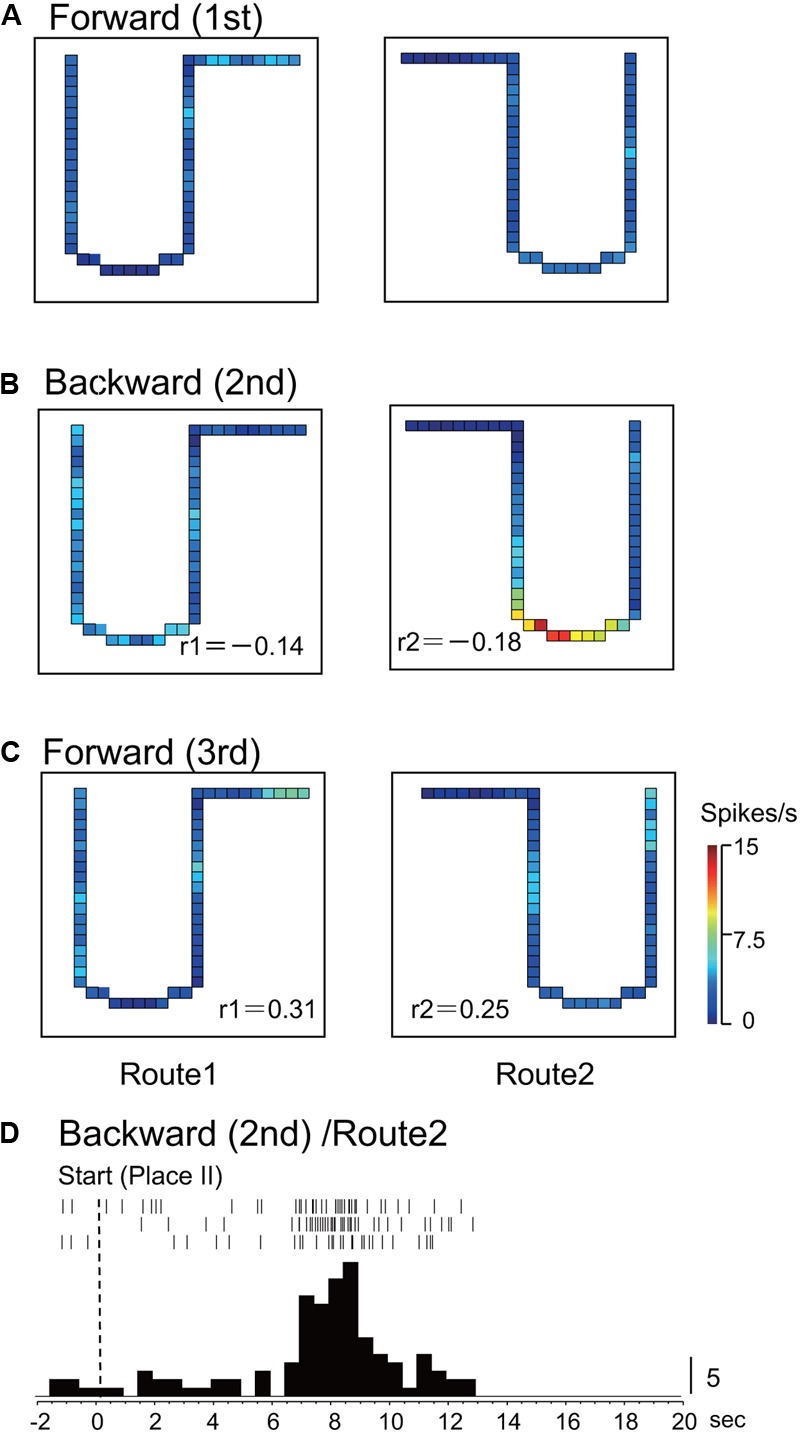
An example of a backward-related place-differential activity. **(A–C)** This activity displayed a place field in Route 2 only in the backward session **(B)**, but not in the forward sessions **(A,C)**. **(D)** Peri-event time histogram of the neuronal activity during backward translocation in Route 2 in the 2nd session. The neuronal activity increased around 8 s after the start from Place II. Other conventions are the same as in **Figure 2**.

**FIGURE 4:**
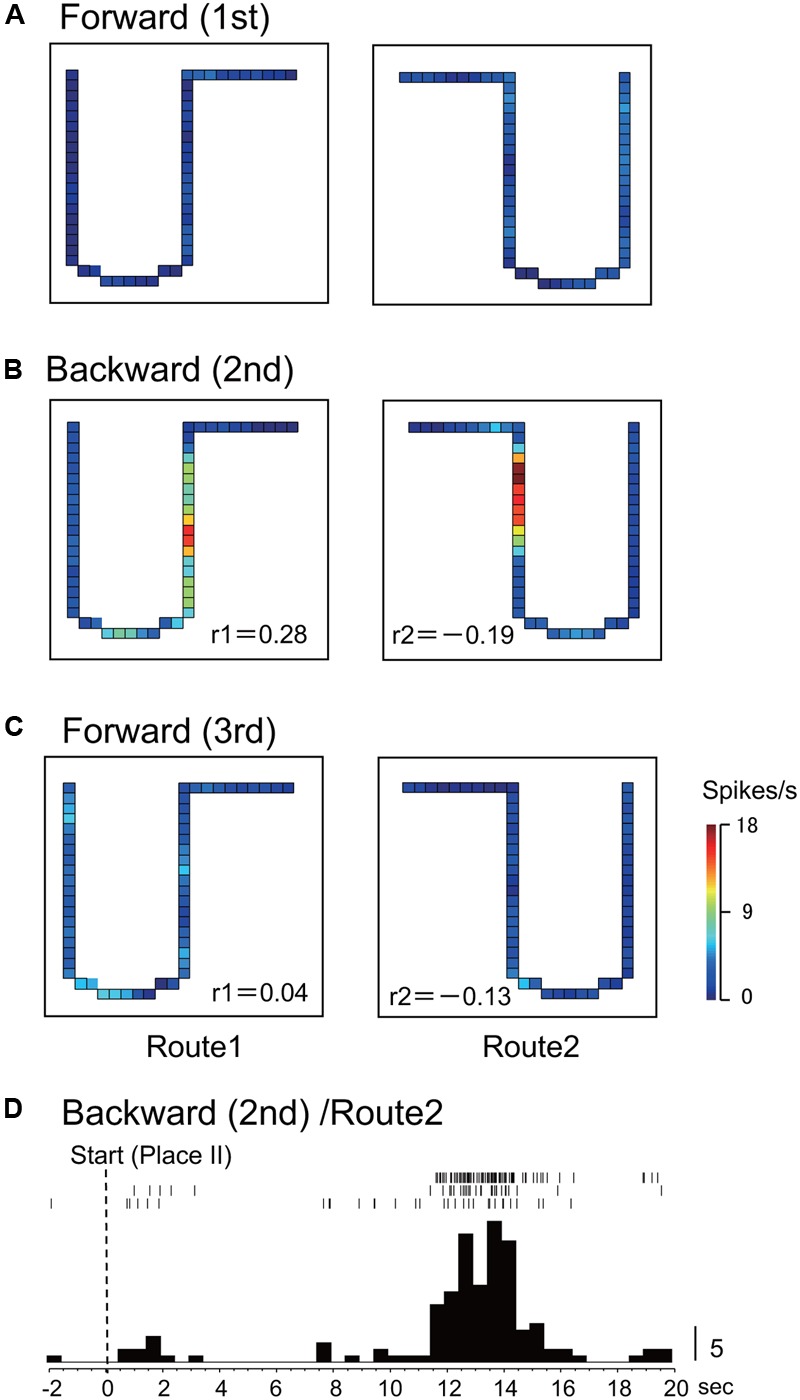
Another example of a backward-related place-differential activity. **(A–C)** This activity displayed place fields in both Routes 1 and 2 only in the backward session **(B)**, but not in the forward sessions **(A,C)**. **(D)** Peri-event time histogram of the neuronal activity during backward translocation in Route 2 in the 2nd session. The neuronal activity increased around 13 s after the start from Place II. Other conventions are the same as in **Figure 2**.

In Route 1, 14 activities (25.0%) did not display significant place fields in the 1st and the 3rd forward sessions, but place fields appeared in the backward session. In Route 2, 5 activities (8.9%) did so in the same way. **Figures 3, 4** illustrate the examples of HF place-differential activities that displayed place fields in Route 2 (**Figure 3**) and the common stem of Routes 1 and 2 (**Figure 4**) only in the backward session, respectively. Since these activities displayed place fields only in the 2nd session, the correlation coefficients between the 1st and the 2nd sessions were low [-0.18 (Route 2) in **Figure 3**; 0.28 (Route 1) and -0.19 (Route 2) in **Figure 4**]. Examples of peri-event time histograms of the neuronal activity during backward translocation in Route 2 in the 2nd session is shown in **Figures 3D, 4D**. The neuronal activity increased around 8 and 13 s after the start from Place II in **Figures 3D, 4D**, respectively.

### Both-Translocation-Related Activities

In Route 1, 11 activities (19.6%) displayed place fields in each session. Furthermore, Pearson’s correlation coefficients between the 1st and the 3rd forward sessions as well as those between the 1st forward and the 2nd backward sessions were larger than 0.4 in these activities. In Route 2, 3 activities (5.3%) displayed the place fields in the same way. The mean correlation coefficients (0.61 ± 0.03) between the 1st and the 3rd forward sessions were comparable to those (0.6 ± 0.04) between the 1st and the 2nd session (Wilcoxon signed rank sum test, *P* > 0.05).

**FIGURE 5:**
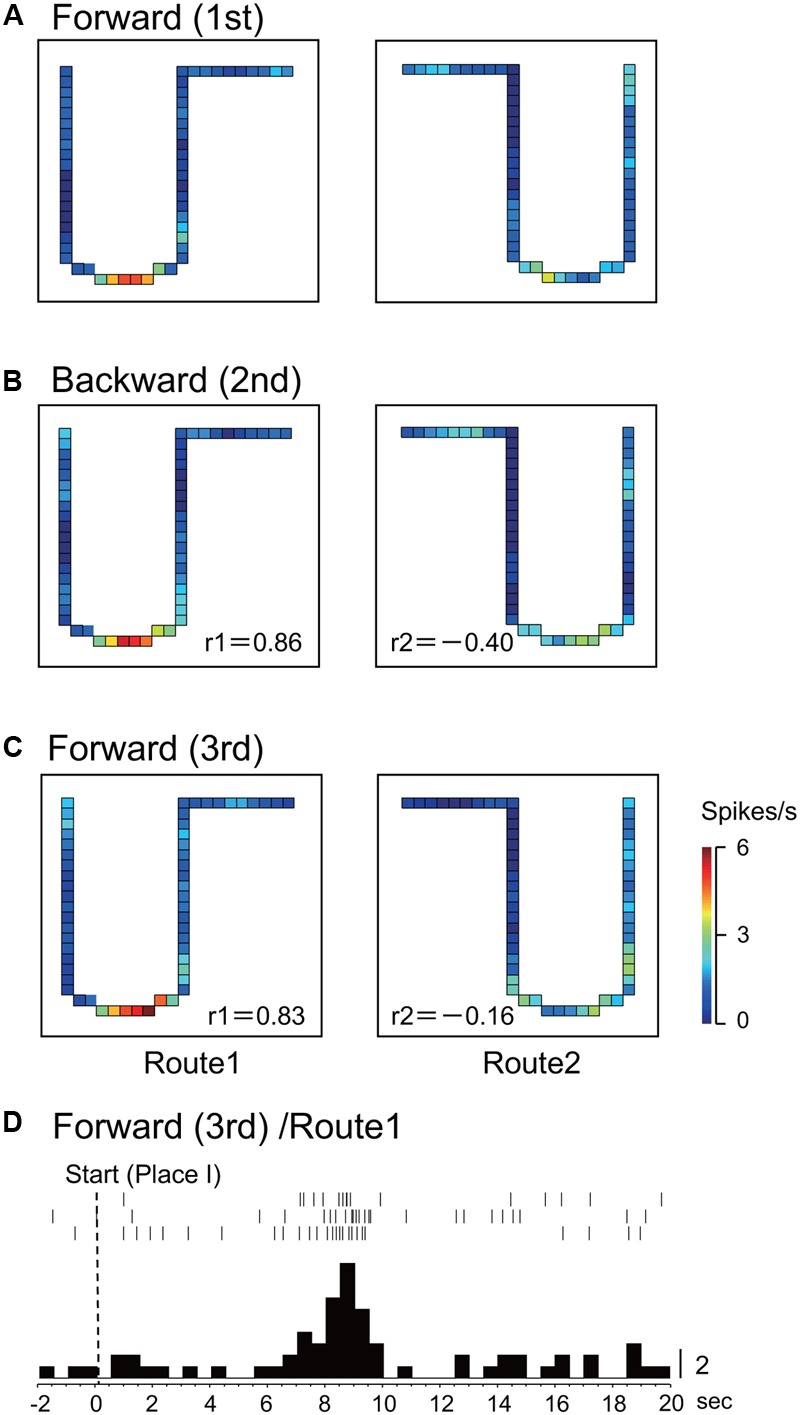
An example of a both-translocation-related place-differential activity. **(A–C)** The place fields were consistently observed across the three sessions in Route 1. **(D)** Peri-event time histogram of the neuronal activity during forward translocation in Route 1 in the 3rd session. The neuronal activity increased around 9 s after the start from Place I. Other conventions are the same as in **Figure 2**.

**Figure 5** illustrates an example of a place-differential activity showing stable place fields in the three sessions in Route 1. This activity displayed place fields in both the 1st and the 2nd sessions. Furthermore, the place fields did not change across the sessions (*r* = 0.86, 1st forward vs. 2nd backward sessions; *r* = 0.83, 1st and 3rd forward sessions). An example of a peri-event time histogram of the neuronal activity during forward translocation in Route 1 in the 3rd session is shown in **Figure 5D**. The neuronal activity increased around 9 s after the start from Place I.

### Session-Dependent Activities

**FIGURE 6:**
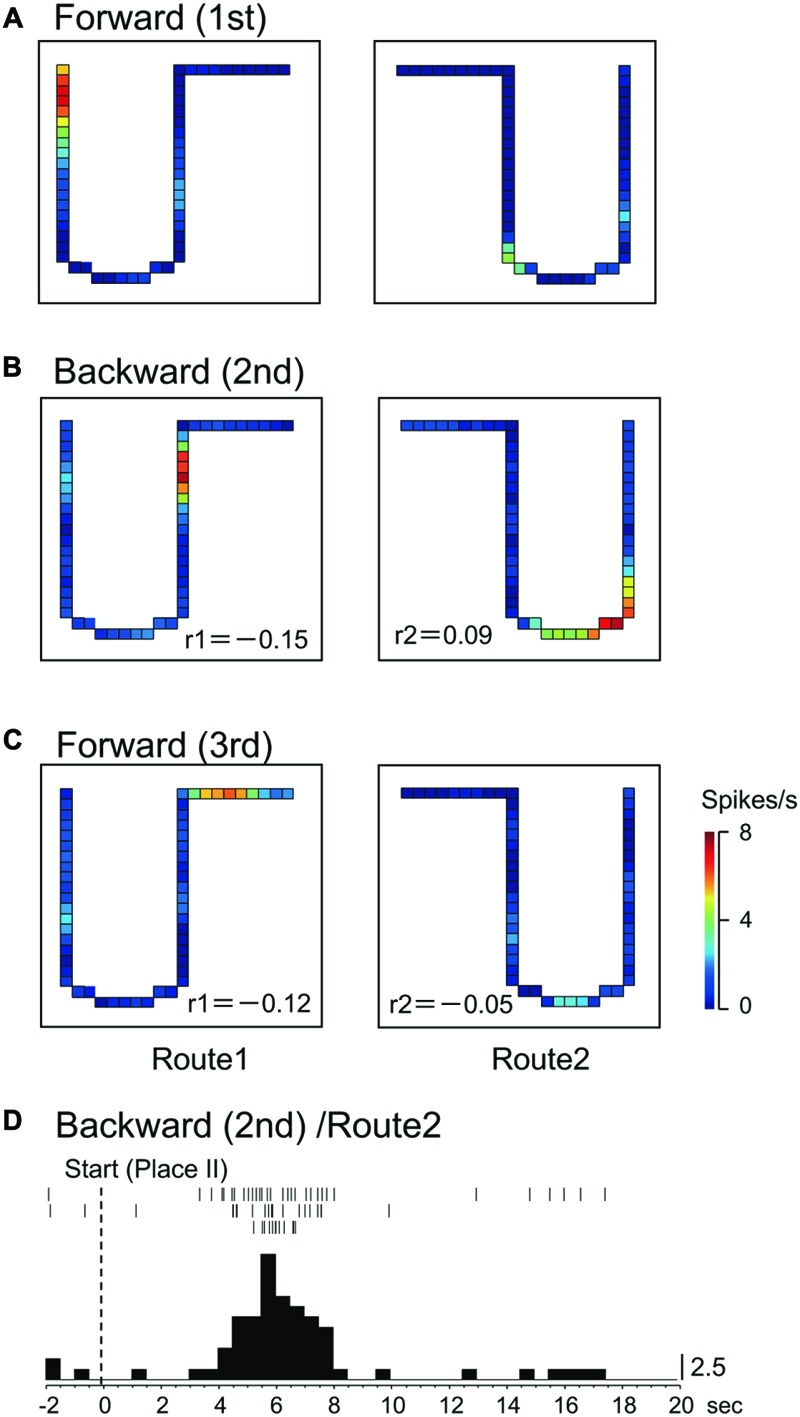
A HF place-differential activity with session-dependent spatial firing patterns under the three sessions in Route 1 (session-dependent). **(A–C)** This activity also displayed backward-related spatial firing patterns in Route 2. **(D)** Peri-event time histogram of the neuronal activity during backward translocation in Route 2 in the 2nd session. The neuronal activity increased around 6 s after the start from Place II. Other conventions are the same as in **Figure 2**.

Of these 56 place-differential activities, 12 (21.4%) showed session-dependent spatial firing patterns, which remapped the place field, in both routes across sessions. The other 6 (10.7%) activities showed a session-dependent spatial firing pattern across sessions in Route 1, while other 15 (26.8%) showed session-dependent spatial firing patterns across sessions in Route 2. **Figure 6** illustrates an example of a place-differential activity showing session-dependent place fields in Route 1. The activity displayed different place fields across the sessions, which resulted in low correlation coefficients between the 1st forward and the 2nd backward sessions (*r* = -0.15), and between the 1st and 3rd forward sessions (*r* = -0.12). However, this activity also displayed a place field only in the 2nd backward session in Route 2 (backward-related activity). An example of a peri-event time histogram of the neuronal activity during backward translocation in Route 2 in the 2nd session is shown in **Figure 6D**. The neuronal activity increased around 6 s after the start from Place II.

### Comparison of the Place Fields

**FIGURE 7:**
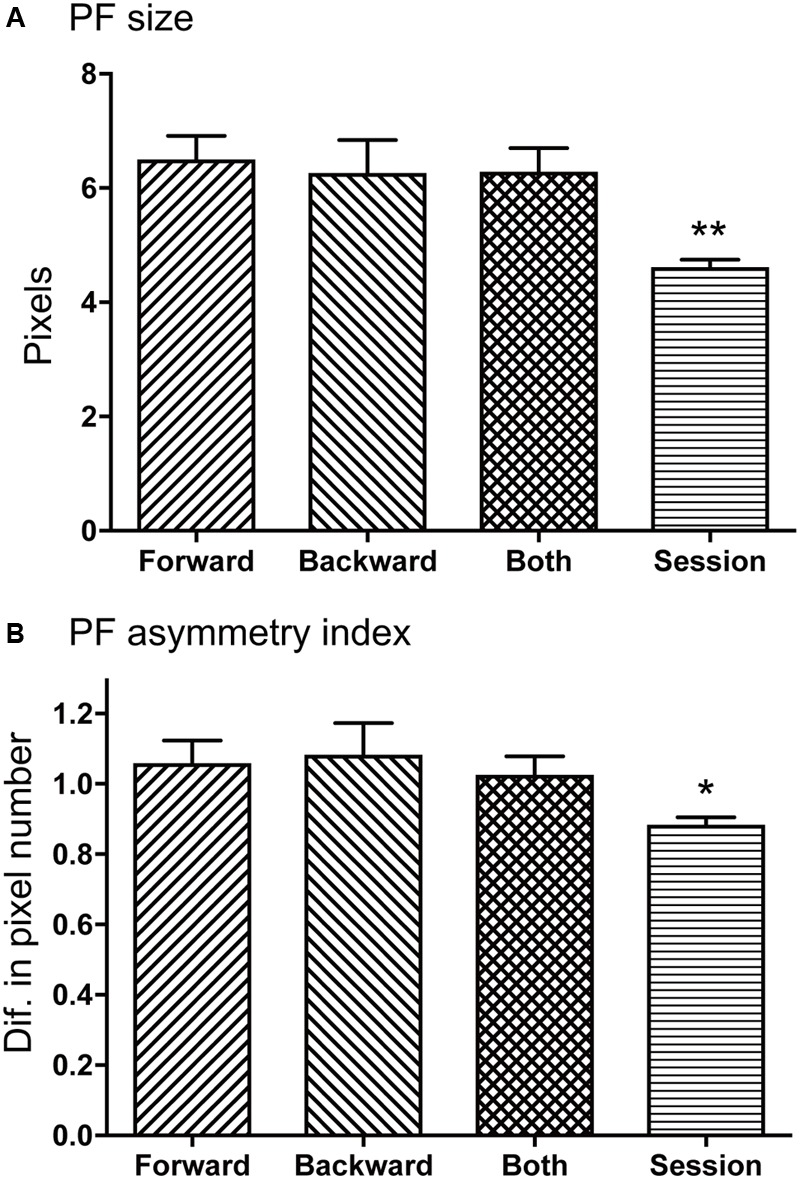
Comparison of mean place field sizes **(A)** and place field asymmetry indices **(B)** among the four types of the HF place-differential activities. **(A,B)** The mean place field sizes were larger in the forward-related, backward-related, and both-translocation-related activities than that of session-dependent activities **(A)**, while the mean place field asymmetry indices were larger in the forward-related, backward-related, and both-translocation-related activities than that of session-dependent activities **(B)**. Ordinate in B indicates difference in number of pixels. PF, place field; Forward, forward-related activities; Backward, backward-related activities; Both, both-translocation-related activities; Session, session-dependent activities. ^∗∗^, ^∗^ significant difference from the other three columns by *P* < 0.01 and *P* < 0.05.

**Figure 7A** illustrates mean sizes of the place fields per route in each type of the place-differential activities. One way analysis of variance (ANOVA) indicated that there was a significant difference among the groups [*F*(3,248) = 12.98, *P* < 0.001]. *Post hoc* tests by Fisher LSD test indicated that the mean place field sizes were larger in the forward-related, backward-related, and both-translocation-related activities than that of session-dependent activities (*P* < 0.01). In the analysis of skewness, most place-differential activities showed negative values; mean skewness in each type was -0.66 ± 0.08 (forward-related), -0.50 ± 0.07 (backward-related), -0.4995 ± 0.07 (both-translocation-related), and -0.63 ± 0.05 (session-dependent), respectively. However, there was no significant difference in skewness among the 4 types [*F*(3,248) = 1.008, *P* > 0.05].

**Figure 7B** illustrates mean place field asymmetry index for each type of the place-differential activities. One way analysis of variance (ANOVA) indicated that there was a significant difference among the groups [*F*(3,248) = 5.160, *P* < 0.02]. *Post hoc* tests by Fisher LSD test indicated that the mean place field asymmetry indices were larger in the forward-related, backward-related, and both-translocation-related activities than that of session-dependent activities (*P* < 0.05). Positive values of place field asymmetry indices indicate that the center of mass of firing distribution was located in the latter half of the place fields according to its definition and the firing rate gradually increased from the entrance of the place field until the center of the mass, then relatively and suddenly returned to the baseline level when the rat left the place field.

### Locations of Place-Differential Activities

**FIGURE 8:**
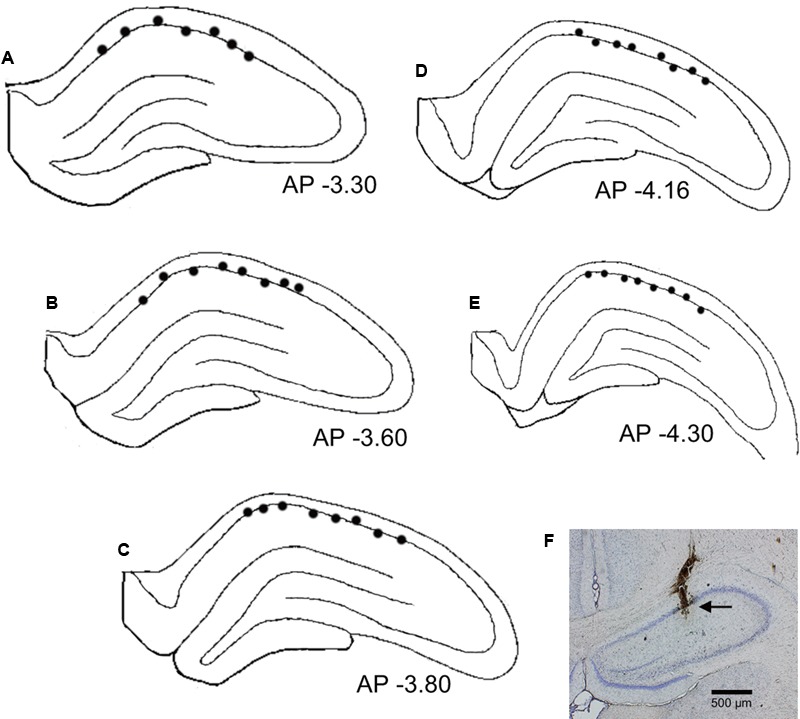
Recording sites of the HF neurons with place-differential activities. **(A–E)** Locations of individual neurons with place-differential activities (filled circles) in each HF section. All HF neurons with place-differential activities were located in the CA1 subfields of the HF. Negative value below each section indicates distance (mm) posterior to the bregma. **(F)** An example of a coronal histological section with an electric lesion (black arrow) in the CA1 area.

**Figures 8A–E** shows recording sites of the place-differential activities. These sites were stereotaxically computed from small lesions made in the HF after recording. An example of a lesion is shown in **Figure 8F**. All the HF neurons with place-differential activities were located in the CA1 area.

## Discussion

We investigated whether HF place cells could encode new configuration of conflicting sensory inputs after repeated exposure to the conflicting environment. Consistent with our hypothesis that the differences in spatial responses between the two studies (Cei et al., 2014; Maurer et al., 2014) might be attributed to the differences in experiences (training) in the backward condition, we found forward- and backward-related activities that were active only in the forward and backward translocation in rats with repeated experience, respectively, although locomotion was the same in these two translocations. Furthermore, the place fields of these HF activities showed experience-dependent changes in skewness. These results are discussed in terms of learning-related synaptic modification by repeated experience (see below).

### Characteristics of HF Place-Differential Activities

In the present study, 4 main types of HF spatial firing patterns were observed when rats were translocated forward and backward. It should be emphasized that the rats were well-trained for more than 5 days in the backward sessions. Our previous study, which used the same set up, indicated that theta activity in the backward sessions decreased to a level comparable to that of the initial forward sessions after training for 5 days (Zou et al., 2009). These findings strongly suggest that the rats adapted well to the mismatched condition, which further suggests that these four types of neuronal activities were not mismatch cells, but rather place cells encoding a new configuration of sensory inputs. Furthermore, these place-differential activities displayed characteristics of learning-related modification in the place fields (see below in detail).

The detailed analyses of the place fields indicated that the place fields were asymmetric; skewness of the place fields was negative, and the place field asymmetry index was positive. It is noted that these asymmetric changes were consistent with the movement direction of the motion stage even in the conflicting condition (i.e., backward-related activities) in the present study. These patterns of firing distributions were similar to those reported in previous studies (Mehta et al., 1997, 2000; Lee et al., 2004), in which the size of place fields expanded by repeated experience in a direction opposite to the rat’s movement. Since NMDA-dependent and temporally asymmetric long-term potentiation/long-term depression (LTP/LTD) is implicated in these types of place field changes (Mehta et al., 1997, 2000; Ekstrom et al., 2001; Lee et al., 2004), the present results suggest that HF neural circuits for the forward-related, backward-related, and both-translocation-related activities might be also modified by these NMDA-dependent and temporally asymmetrical processes. Consistent with this idea, a cross-correlation study of monkey HF neurons reported that information of spatial navigation in specific tasks is encoded by the temporally asymmetrical neural circuits connecting pyramidal neurons (Hori et al., 2011).

Our previous neurophysiological study using the same experimental set up reported that these place-differential activities were dependent on locomotion (proprioceptive and/or motor efferent copy), the task context, and vestibular sensation or visual cues such as optic flow (Dayawansa et al., 2006). These findings suggest that the forward-related and backward-related place-differential activities might play a role in encoding configuration of convergent sensory inputs (optic flow, vestibular inputs, proprioceptive and/or motor efferent copy) in the forward and backward sessions, respectively. In the present study, the activity of both-translocation-related activities increased in the same place in both the forward and backward sessions, where optic flow and vestibular sensation conflicted. These activities might encode location of the animals based on distal cues regardless of other sensations (optic flow, vestibular, and proprioceptive sensation). We also observed some HF activities that showed session-dependent spatial firings in each session, consistent with our previous study (Dayawansa et al., 2006). Consistent with the present results, another previous study indicated that the place cells sometimes spontaneously remapped in a stable environment (Ludvig, 1999). Taken together, these results suggest that the HF can encode and predict future movements that do not occur in a natural environment by repeated experience.

### Effects of Learning-Related Synaptic Modification on Neural Correlates to Space

There were significant differences among the four types of place-differential activities; place field sizes were larger in the forward-related, backward-related, and both-translocation-related activities than in the session-dependent activities, and the place field asymmetry index also showed a similar trend. These findings suggest that repeated training-related changes in the place fields are more evident in the forward-related, backward-related, and both-translocation-related activities than in the session-dependent activities. These differences in training-related changes (place field asymmetry index, place field size) may account for the difference in stability of the place fields among the four types of the HF place-differential activities (see below in detail).

It is reported that stability of place cell firing fields depended on attentional demands to spatial landmarks; when the animals freely explore an environment under no task contingencies, place fields were not stable (Kentros et al., 2004; Muzzio et al., 2009). This attentional modulation was mediated through dopaminergic D1/D5 receptors (Kentros et al., 2004), and activation of D1 receptor upregulates NMDA receptor-mediated LTP (Nai et al., 2010). In the present experimental set up, the rats were translocated by the motion stage, and as a consequence, they might not strongly attend to the external landmarks. Therefore, the place fields of some HF neurons with session-dependent activities might not be stabilized by attentional modulation. Consistent with this idea, the mean place field sizes and place field asymmetry indices, which are dependent on NMDA-dependent LTP (Ekstrom et al., 2001), were smaller in the session-dependent activities. These results suggest that HF neural circuits for the forward-related, backward-related, and both-translocation-related activities might be more extensively modified by these NMDA-dependent LTP/LTD and consequently more stabilized than those for the session-dependent activities. The lack of stabilization by these NMDA-dependent processes in the session-dependent activities might account for the unstable firing distributions across the sessions. On the other hand, it is reported that HF neuronal activity gradually changes even in the same environment by encoding temporal context (Manns et al., 2007a). This mechanism might also contribute to spatial remapping in session-dependent activities.

There are some discrepancies among the studies reporting place field shifts. Some previous studies reported that the center of the mass of the place fields shifted backward after repeated trials (Mehta et al., 1997, 2000; Lee et al., 2004). However, other studies reported opposite changes; forward shift of the center of the mass of the place fields (Lee et al., 2006; Griffin et al., 2007). Nevertheless, skewness was consistently negative in all of the previous studies including the present study (i.e., the firing rates gradually increase, but abruptly decrease). These results strongly suggest that the HF neural circuits are subject to certain learning-induced synaptic modification, consistent with the recent studies (Cheng and Frank, 2008; Komorowski et al., 2009; Tort et al., 2009; Wirth et al., 2009; Lever et al., 2010). The modes of these neural activity changes due to training-induced synaptic modification might depend on task demands for future prediction. The HF might be essential to encode the past events to form episodic memory, and based on such memory the HF is also essential to predict goals, future trajectories, and outcome of the events that have not yet occurred (Eichenbaum and Fortin, 2009; Takahashi, 2013, 2015). Some HF activities with backward shifts of the place fields in the present as well as previous studies (Mehta et al., 1997, 2000; Lee et al., 2004) might be involved in prediction of future location, while the HF neurons with forward shifts of the place fields (Lee et al., 2006; Griffin et al., 2007) might be involved more in reward prediction in which place field is expanded to future reward location. This difference in prediction demands might result in different shifts of the place fields among the studies. A recent study suggests that prediction and future planning might be executed by the interaction between the HF CA1 area and medial prefrontal cortex (Ishino et al., 2017).

### Comparison with the Head-Direction Cell System

The head-direction cell system (Taube et al., 1990a,b; Taube, 1995) integrates head angular velocity to output a signal related to head direction (McNaughton et al., 1991; Blair and Sharp, 1995; Skaggs et al., 1995; Zhang, 1996). Movement direction on a radial maze strongly influences HF place cell activity (McNaughton et al., 1983). Firing properties of HF place cells are related to head-direction cell activity (Knierim et al., 1995), and the head-direction system might orient the ‘cognitive map’ in the HF (O’Keefe and Nadel, 1978; Knierim et al., 2000). Several studies have analyzed the activity of head direction cells when an animal was placed in a specific situation with conflicting spatial information. For example, activity of head-direction cells in the anterior dorsal thalamic nucleus was recorded under conditions where vestibular cues conflicted with optic flow (Blair and Sharp, 1996). In this mismatch condition, the majority of the cells were bound to the vestibular information. This suggests that vestibular information is essential in the neural computation for head direction (Taube, 1998). On the other hand, in well-trained rats in the same setup as in the present study, we reported four types of thalamic neurons (Enkhjargal et al., 2014); heading direction-related and movement direction-related neurons coded separately heading and movement directions regardless of direction of translocation (forward or backward), while forward and backward movement-related neurons coded movement directions only in forward and backward translocation, respectively. These results suggest that thalamic neurons can encode conflicting multiple information to identify heading and movement directions in a backward condition, if animals are well-trained.

In the present study, some HF activities (backward-related and both-translocation-related activities) were able to encode conflicting sensory/motor information in the backward session, where vestibular and visual information did not match proprioceptive information (or motor efferent copy). These results strongly suggest that HF neurons encode configuration of convergent sensory inputs, in which vestibular information is one of the sensory inputs, but not a major input. This demonstrates that the HF encodes any combination of the sensory inputs. Since HF lesions affect head direction cell activity in the thalamus (Golob and Taube, 1999), HF outputs might be integrated in the anterior thalamus (Aggleton et al., 2010). Therefore, configural information from the HF may contribute to the complex responsiveness of thalamic neurons in a well-trained familiar condition.

### The Role of the HF in Adaptation to Mismatch Conditions

Various situations inducing sensory mismatch by vection and microgravity in space ships provoke motion sickness with autonomic disturbances in approximately 60% of both healthy subjects and astronauts (Stern et al., 1989). Previous human and animal studies indicated that HF activity was associated with autonomic functions or visceral sensation (Jasper and Rasmussen, 1958; Van Buren, 1963; Pedemonte et al., 2003). Furthermore, the HF sends dense afferent fibers to the autonomic centers including the hypothalamus and amygdala (see a review by Petrovich et al., 2001). These findings suggest that autonomic disturbances in motion sickness might be induced partly by alteration of HF activity induced by novel sensory conflict.

It has been reported that training ameliorates symptoms of motion sickness in subjects susceptible to vection-induced motion sickness (Stern et al., 1989). Furthermore, an artificially generated environment for orientation and motion with similar sensory-conflicts in space is effective for preflight training, which might be mediated through habituation process, and has been used to treat motion sickness (Clemens and Howarth, 2003). Consistent with these clinical studies, repeated exposure to a conflicting condition (backward translocation with locomotion) decreased HF theta activity (Zou et al., 2009). In the present study, some HF place-differential activities encoded sensory information in backward translocation after repeated exposure to this condition. Another study, in which HF place cells were recorded from the rats traversing a 3-dimensional track consisting of the three surfaces during the Neurolab Space Shuttle mission, reported that location-specific firing was initially abnormal or poor, but later became specific to a single surface of the track (Knierim et al., 2000, 2003). These findings suggest that the formation of new place cells encoding new conflicting environmental information might contribute to the updating of stored memory in the HF, enabling a HF comparator to accept this new environment as a match with the stored information.

Furthermore, the present results indicate that the forward-related and backward-related HF activities showed place fields only in specific sessions without any changes in environmental cues. Consistent with this result, cognitive requirements or task paradigms influence place cell activity even in the same environment (Markus et al., 1995; Frank et al., 2000; Wood et al., 2000; Smith and Mizumori, 2006a,b; Takahashi, 2013, 2015). A human behavioral study reported that some subjects were able to adapt context-dependently to different conditions, in which visual information mismatched proprioceptive-vestibular inputs in a virtual environment (Dumontheil et al., 2006). These findings suggest that the HF is important for context-dependent adaptation.

## Conclusion

The present results indicate that the HF place cells have the ability to encode a new configuration of sensory inputs, which do not occur naturally, to update information in the HF comparator.

## Author Contributions

HisN conceived the study. HisN designed the experiment. DZ performed the experiment. DZ and HisN analyzed data and wrote the paper. HisN, HirN, JM, YT, and TO revised the paper. All the authors discussed the results and commented on the manuscript, and read and approved the final manuscript.

## Conflict of Interest Statement

The authors declare that the research was conducted in the absence of any commercial or financial relationships that could be construed as a potential conflict of interest.
