# Supplementary material for: Experience-Related Changes in Place Cell Responses to New Sensory Configuration That Does Not Occur in the Natural Environment in the Rat Hippocampus
Source: Front Pharmacol. 2017 Aug 23;8:581. doi: 10.3389/fphar.2017.00581 (PMC5572398; doi:10.3389/fphar.2017.00581)
Supplement: Supplementary file 1 [file Presentation_1.PDF]

Supplementary Material

Experience-related changes in place cell responses to new sensory configuration that does not occur in the natural environment in the rat hippocampus

Dan Zou, Hiroshi Nishimaru, Jumpei Matsumoto, Takamura Yusaku, Taketoshi Ono, Hisao Nishijo

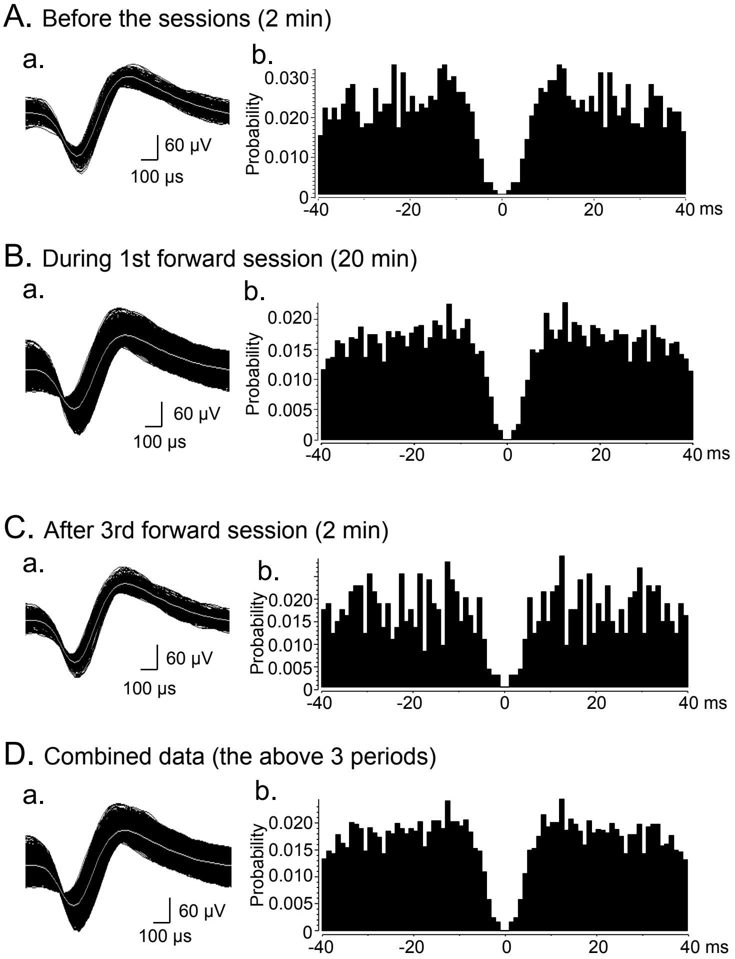

Supplementary Figure 1. Identification of a single neuron in the HF CA1 subfield across the sessions on basis of extracellularly recorded physiological parameters.

A-C: Stable single neuronal activity was checked by drawing superimposed traces of the HF neuron (a) and analyzing autocorrelograms (b) before (A) and during (B) the 1st (forward) session, and after the 3rd (forward) session (C). In b, bin width is 1 ms. The ordinates indicate probability where bin counts were divided by the number of spikes in the spike train. D: Combined data in A, B, and C.
